# Red-Emitting SBBF (Single-Benzene-Based Fluorophore)-Silica Hybrid Material: One-Pot Synthesis, Characterization, and Biomedical Applications

**DOI:** 10.3390/nano11082036

**Published:** 2021-08-10

**Authors:** Jaehoon Kim, Jong Min An, Yuna Jung, Na Hee Kim, Youngwoong Kim, Dokyoung Kim

**Affiliations:** 1 Department of Biomedical Science, Graduate School, Kyung Hee University, Seoul 02447, Korea; noblestylejay@gmail.com (J.K.); jmahn1990@gmail.com (J.M.A.); jungpeng159@gmail.com (Y.J.); pionaheek@gmail.com (N.H.K.); eeewq1111@gmail.com (Y.K.); 2 Department of Anatomy and Neurobiology, College of Medicine, Kyung Hee University, Seoul 02447, Korea; 3 Center for Converging Humanities, Kyung Hee University, Seoul 02447, Korea; 4 Medical Research Center for Bioreaction to Reactive Oxygen Species and Biomedical Science Institute, Kyung Hee University, Seoul 02447, Korea; 5 KHU-KIST Department of Converging Science and Technology, Kyung Hee University, Seoul 02447, Korea

**Keywords:** single-benzene fluorophore, hybrid material, red-emitting, bio-imaging, antibiotic

## Abstract

We report, for the first time, a new red-emitting hybrid material based on a single-benzene-based fluorophore (SBBF) and silica. This robust formulation shows several features, including bright emissions at a red wavelength (>600 nm), high scalability (>gram-scale), facile synthesis (one-pot reaction; SBBF formation, hydrolytic condensation, propagation), high stability (under different humidity, pH, light), bio-imaging applicability with low cellular toxicity, and an antibacterial effect within Gram-negative/Gram-positive strains. Based on our findings, we believe that these hybrid materials can pave the way for the further development of dye-hybrid materials and applications in various fields.

## 1. Introduction

Fluorescent materials have been widely used throughout various fields over the last century, including in basic research, industry, and translational medicine [1,2,3,4]. Fluorescent proteins and small organic fluorophores, in particular, have ushered in a new era in chemical biology and materials science [5,6,7,8]. To date, hundreds of organic fluorophores have been reported, and many studies have disclosed novel rationales for the molecular structure design with their unique applications such as molecular sensing, disease biomarker analysis, bio-imaging, drug discovery, and so on [9].

Recently, scientists have been interested in the development of hybrid materials between the organic fluorophores and inorganic ingredients in the hope of the following: (i) enhanced physicochemical property, (ii) enhanced stability under harsh environments, (iii) enhanced molecular sensing abilities, (iv) enhanced biocompatibilities, and (v) targeted bio-imaging of specific cells or disease sites [10,11,12].

**Figure 1:**
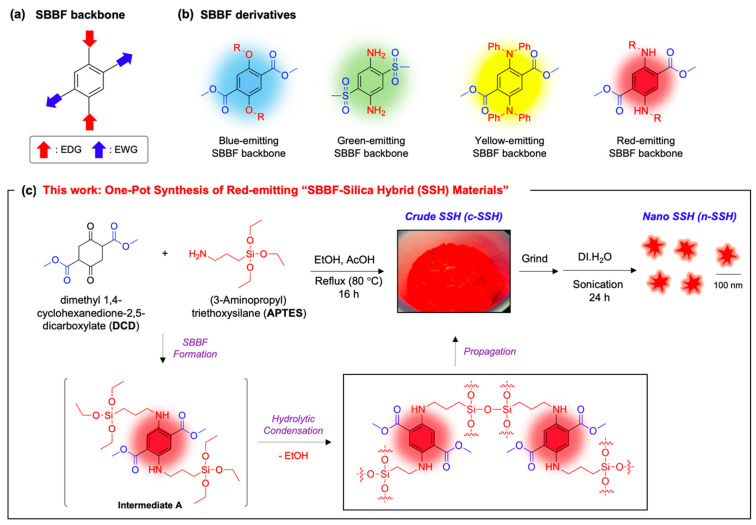
SBBF backbone, derivatives, and **SSH** materials. (**a**) Chemical structure of SBBF (single-benzene-based fluorophore) backbone. EDG: electron-donating group. EWG: electron-withdrawing group. (**b**) Structures of blue-/green-/yellow-/red-emitting SBBF derivatives. (**c**) This work: one-pot synthesis of red-emitting SBBF-silica hybrid (**SSH**) materials; crude **SSH** (**c-SSH**), nano-sized **SSH** (**n-SSH**).

As a research group within the field of fluorescence-based molecular sensing and bio-imaging [13,14,15], we have focused on the development of new SBBF (single-benzene-based fluorophore) derivatives [16,17] and its hybrid materials with inorganic ingredients (e.g., silica, silicon, calcium silicate). The SBBF has an electron-donor (D)-acceptor (A) type dipolar structure (Figure 1a), and its emission property could be facilely tunable by changing the functional group at the donor or acceptor site (see Figure 1b for representative SBBF derivatives) [18,19,20,21,22]. The SBBF has some notable features compared to other well-known organic fluorophores: (i) compact size, (ii) facile wavelength tuning, (iii) bright blue-/green-/red-emission in solid-/solution-state, and (iv) polarity-independent solvatochromic effect. However, the SBBF has its drawbacks, such as a pH-dependent emission, low stability under light irradiation, and limited biological applicability, which need to be overcome through the introduction of a new molecular or material concept.

In this present work, we disclose a new hybrid formulation of SBBF and silica for the first time (Figure 1c). The SBBF-silica hybrid (**SSH**) materials showed several features: (i) bright emission at a red wavelength (>600 nm), (ii) high scalability (>gram-scale), (iii) one-pot reaction, whereas previously reported silica hybrid materials used silica nanoparticles or were formed through a complex process [11,12], (iv) high stability, (v) bio-imaging applicability with low cellular toxicity, and (vi) antibacterial effect with Gram-negative/Gram-positive strains. The preparation and characterization of the **SSH** along with their physicochemical properties were systematically identified, and their biomedical applications, including cellular imaging and bacteria-killing, were successfully demonstrated. The present findings hold great promise for the application of **SSH** in basic science as well as in biomedical fields.

## 2. Materials and Methods

### 2.1. Materials

Dimethyl 1,4-Cyclohexanedione-2,5-dicarboxylate (DCD) was purchased from TCI (Tokyo, Japan), (3-aminopropyl)-triethoxysilane (APTES) was purchased from Sigma-Aldrich (Burlington, MA, USA), and 1-propylamine was purchased from Alfa Aesar (Ward Hill, MA, USA). Organic solvents, including ethanol, dimethyl sulfoxide, n-Hexane, acetonitrile, ethyl acetate, acetone, tetrahydrofuran, dichloromethane, and sodium chloride were purchased from Samchun (Seoul, Korea). The cellular sub-organelle imaging agents (cell mask green plasma membrane stain, 4′,6-diamidnino-2-phenylindole (DAPI)) were purchased from ThermoFisher (US). Dulbecco’s modified Eagle’s media (DMEM) and fetal bovine serum (FBS) for cell culture (HEK293) were purchased from Hyclone (Logan, UT, USA). The pH buffers were purchased from Daejung Chemicals & Metals co., LTD (Siheung, Korea). The cell culture dish (SPL Life Science, #20060, Pocheon, Korea) was purchased for the applications. Commercially available reagents and anhydrous solvents were used without further purification. Chemical reactions were performed in open-air reflux conditions. ^1^H NMR spectra were recorded on a JNM-ECZ500R (500 MHz, JEOL, Tokyo, Japan). In the NMR spectra, the chemical shifts (δ) were reported in ppm, and the multiplicities are indicated by s (singlet), d (doublet), t (triplet), dd (doublet of doublets), and m (multiplet). Coupling constants were reported in Hz. Chemical shifts were reported in parts per million (ppm) and measured relative to the signal (0.00 ppm) of internal tetramethylsilane (TMS) in CD_3_CN (1.94 ppm). Cell imaging was conducted using a confocal laser scanning microscope (CLSM, LSM-800, Carl Zeiss, Oberkochen, Germany). The confocal images of **n-SSH** (red), cell mask (green), and DAPI (blue) in HEK293 cells were obtained under the excitation at 561, 488, and 405 nm (Laser power: 0.80, 0.20, and 3.50%) with a detector (GaAsP, Detector Gain: 700, 650, and 650 V; Detection wavelength: 576–700, 513–546, and 410–450 nm). The fluorescence intensity of the materials was measured with a fluorescence imaging system (FTIS, VISQUE^®^ InVivo Elite, Vieworks Co., Ltd., Anyang, Korea).

### 2.2. Preparation of **c-SSH**

APTES (3 eq) was added to the ethanol solution (5 mL) containing DCD (0.4382 mmol) and acetic acid (50 μL, 2 eq). The mixture was then refluxed for 18 h under an open-air atmosphere [23]. After the reaction, the mixture was cooled down to room temperature and filtrated. Red-color solid compound (250 mg) was afforded and rinsed with deionized water (DI.H_2_O) and acetone three times. The yield was 90.2% after the washing step.

### 2.3. Characterization of **c-SSH**

The surface element of the particles was analyzed using energy-dispersive X-ray spectroscopy (EDS, E-max Evolution EX-370 Analyzer) at the Korea Basic Science Center (Korea University, Seoul, Korea). Attenuated total reflection-Fourier transform infrared (ATR-FTIR) spectroscopy was performed using a Thermo Scientific Nicolet™ iS™ 5 FT-IR spectrometer instrument (16 scans, Waltham, MA, USA). The thermal stability of **c-SSH** and DCD was evaluated using thermogravimetric analysis (TGA, SDT Q600, TA Instruments, New Castle, DE, USA) at Hanyang University (Seoul, Korea). The fluorescence changes at different humidities and temperatures were measured using FTIS (VISQUE^®^ InVivo Elite, Vieworks Co., Ltd., Korea). Time-resolved fluorescence (TRF) signals of **c-SSH** were measured in solid-state. Experiments were carried out using a time-correlated single-photon counting (TCSPC) method. The sample was excited using a 520 nm pulse (LDH-P-C-520, PicoQuant), and the sample TRF signals were collected at 610 nm. The TRF signals were measured by using a photomultiplier tube (PMA 182, PicoQuant). The instrumental response function (IRF) of our TRF setup is −120 ps. The average lifetime of **c-SSH** is 3.01 ns. The optimized molecular structure, HOMO/LUMO, and the energy levels of **Intermediate A** were analyzed by DFT calculations (B3LYP-d3/6-31+G(d)).

### 2.4. Preparation of **n-SSH**

**c-SSH** (100 mg) was added into DI.H_2_O (5 mL), within a sealed glass vial, (Volume: 20 mL, VWR, Product No. 66011-143, Radnor, PA, USA) and fractured into nanoparticles in an ultrasonic bath (VWR, PA, USA) for 24 h. The resulting **n-SSH** was sorted using a centrifuge (Eppendorf Centrifuge model 5418, 5418FQ924939, Eppendorf, Hamburg, Germany) at 15,000 rpm for 30 min, and the supernatant was collected. The supernatant contains **n-SSH** (yield: 20%).

### 2.5. Characterization of **n-SSH**

Hydrodynamic size and zeta-potential of **n-SSH** were analyzed by Malvern Instruments Zetasizer Nano ZS90 (Worcestershire, UK). The structural morphologies of **n-SSH** were visualized by transmission electron microscopy (TEM, Tecnai, G2 F30ST, FEI Company, Hillsboro, OR, USA) and scanning electron microscope (SEM, SU8220, Hitachi, Japan) imaging at the Korea Basic Science Center (Korea University, Seoul, Korea). The surface element of particles was analyzed using energy-dispersive X-ray spectroscopy (EDS, E-max Evolution EX-370 Analyzer) at the Korea Basic Science Center (Korea University, Seoul, Korea).

### 2.6. Preparation of **DCD-propyl**

1-propylamine (4 eq) was added to the ethanol solution (5 mL) containing DCD (0.219 mmol) and acetic acid (50 μL, 2 eq). The mixture was refluxed for 18 h under an open-air atmosphere and then was cooled down to room temperature and filtrated [23]. Red solid compound (50 mg) was obtained and rinsed three times with deionized water (DI.H_2_O) and acetone. ^1^H NMR spectra were recorded on a JNM-ECZ500R (500 MHz, US). In the ^1^H NMR spectra, the chemical shifts (δ) are reported in ppm, and multiplicities are indicated by s (singlet), d (doublet), t (triplet), dd (doublet of doublets), and m (multiplet). Coupling constants were reported in Hz. Chemical shifts were reported in parts per million (ppm) and measured relative to the signal (0.00 ppm) of internal tetramethylsilane (TMS) in CD_3_CN (1.94 ppm). ^1^H-NMR (500 MHz, Acetonitrile-D3) information of **DCD-propyl**: δ 7.28 (s, 2H), 6.72 (s, 2H), 3.84 (s, 6H), 3.10 (q, J = 6.5 Hz, 4H), 1.65 (td, J = 14.5, 7.3 Hz, 4H), 0.99 (t, J = 7.4 Hz, 6H).

### 2.7. UV/vis Absorption and Fluorescence Spectroscopic Methods

UV/vis absorption spectra were measured using a spectrophotometer (Agilent Technologies Cary 8454, Santa Clara, CA, USA), and fluorescence spectra were recorded on a spectro-fluorophotometer (SHIMADZU CORP. RF-6000, Kyoto, Japan), with a 1 cm standard quartz cell (internal volume of 1 mL, Hellma Analytics, Müllheim, Germany). All absorption and emission spectra were obtained at room temperature (25 °C). 

### 2.8. Cell Experiment

#### 2.8.1. Cell Culture

The human embryonic kidney cell line (HEK293) was obtained from Korean Cell Line Bank (KCLB, Seoul, Korea). Cells were cultured in Dulbecco’s modified Eagle’s media (Hyclone, Logan, UT, USA) supplemented with 10% fetal bovine serum (Hyclone) and 1% penicillin-streptomycin (Gibco, New York, NY, USA). Cell lines were kept in humidified air containing 5% CO_2_ at 37 °C.

#### 2.8.2. Cytotoxicity Analysis

The cytotoxicity of **n-SSH** was evaluated within the HEK293 cell line by a Cell Counting Kit-8 (CCK-8, Dojindo Molecular Tech. Inc, Kumamoto, Japan) assay according to the manufacturer’s protocols. The cells (5 × 10^3^ cells per well) were seeded on 96-well plates and incubated for 24 h at 37 °C. The media was then refilled with fresh media, not containing any serum or antibiotics. The cells were treated with diluted **n-SSH** (0.3125–20 mg/mL) and then incubated for 24 h. After that, the **n-SSH** was removed by washing them with PBS (3 times), followed by changing the serum-free media. CCK-8 solution (10 µL, 10× working concentration) in a serum-free media was added to each well of a 96-well plate, and the cells were incubated for 2 h at 37 °C. After that, the absorbance was measured at a wavelength of 450 nm using a microplate reader (Multiskan FC, Thermo Fisher, Waltham, MA, USA). The percentage of the cell cytotoxicity was calculated using the formula: Cell viability (%) = (Mean OD of sample × 100)/(Mean OD of the control group) (OD: optical density).

#### 2.8.3. CLSM Cellular Imaging

Approximately 1 × 10^5^ cells (HEK293) were seeded onto 35-mm glass confocal dishes and incubated for 24 h. At 80% confluency, the media was replaced with serum-free media. 5 min later, cells were treated with **n-SSH** (10 mg/mL) for 1.5–12 h at 37 °C in a 5% CO_2_ incubator. The cells were washed with PBS (pH 7.4) and treated with organelle tracker in a serum-free media for 20 min at 37 °C in a 5% CO_2_ incubator. [Note: Treatment volume and concentration of sub-organelle tracker: 2 µL (1000× working concentration) of cell mask Green plasma membrane stain and 4′,6-diamidnino-2-phenylindole (DAPI)]. The cells were washed three times with PBS (pH 7.4). After washing, the cells were treated with 4% formaldehyde for 6 min, and the solution was then removed by washing it with PBS (pH 7.4). The prepared samples were imaged using confocal laser scanning microscopy (CLSM, LSM-800, Carl Zeiss, Oberkochen, Germany).

### 2.9. Bacterial Experiment 

#### 2.9.1. Bacterial Strains and Culture

All strain-related studies were conducted in certified BSL-level facilities at Kyung Hee University Medical Center (Seoul, Korea). Strains of drug-sensitive bacteria (control strain) were obtained from the American Type Culture Collection (ATCC, Manassas, VA, USA) and the Culture Collection of Antibiotic-Resistant Microbes (CCARM, Seoul, Korea). Strains of drug-resistant bacteria were obtained from Asan Medical Center (ASM, Seoul, Korea). All bacterial strains were stored in skimmed milk and frozen at −70 °C. The bacterial strains were sub-cultured twice in cation-adjusted Mueller–Hinton broth (CA-MHB) for 24 h at 37 °C, before minimum inhibitory concentration (MIC) analysis and time, or concentration-dependent study.

#### 2.9.2. Minimum Inhibitory Concentration (MIC) Assay

MIC was determined using broth microdilution in CA-MHB according to the Clinical and Laboratory Standard Institute (CLSI, 2016) guidelines. In this study, we performed the MIC assay for 16 types of strains including MDR bacteria. Briefly, **n-SSH**, DCD, DMSO, and CFX were serially diluted (two-fold) using CA-MHB broth in a 96-well microplate. The turbidity of all the strains was adjusted to a 0.5 McFarland standard (1 × 10^8^ CFU/mL) and 10 μL of bacterial suspension was added to each well of a 96-well microplate, with the final concentration of each strain being approximately 5 × 10^5^ CFU/mL. The contents of the microplate were mixed and incubated for 24 h at 37 °C. For the MIC assay, the strain obtained from the microorganism bank was used as the quality control strain. Each experiment was conducted in triplicate.

### 2.10. Statistical Analysis

The results were analyzed by one-way analysis of variance (ANOVA) with Tukey’s multiple comparisons. Data were expressed as mean ± standard error of the mean (S.E.M). All statistical results were analyzed with Prism 8.0 software (GraphPad, La Jolla, CA, USA).

## 3. Results and Discussion

### 3.1. Rational for the Material Design

For the hybridization of the SBBF and silica, we introduced a one-pot synthesis approach, based on an in situ two-step reaction: (i) condensation (SBBF formation) between DCD (dimethyl 1,4-cyclohexanedione-2,5-dicarboxylate) and the APTES (3-aminopropyl triethoxysilane) under an air atmosphere [24], and (ii) hydrolytic condensation and propagation of triethoxysilane moiety under a given reaction condition (solvent: ethanol, temperature: 80 °C). At the design stage of the **SSH** material, we outlined three points: (i) red-emission: the secondary amine moiety at the electron-donating site of the SBBF could generate a red-emission [25], which holds advantages in biological studies [23]; (ii) SBBF-encapsulated silica: the silica wrapping can help to compensate the drawbacks of the SBBF; and (iii) readily scalable and facile synthesis: a simple one-pot reaction using commercially available and inexpensive starting materials.

With these materials, we prepared two different types of **SSH**, crude **SSH** (as-prepared **SSH**; **c-SSH**) and nano-sized **SSH** (**n-SSH**) (Figure 1c). The one-pot synthesis using DCD and APTES gave a red-emitting solid compound named **c-SSH**, and **n-SSH** (particle size: around 100 nm) was prepared by the grinding and sonication of **c-SSH** (See detail methods in the Supplementary Material).

### 3.2. Material Characterization and Photophysical Properties of **c-SSH**

**Figure 2:**
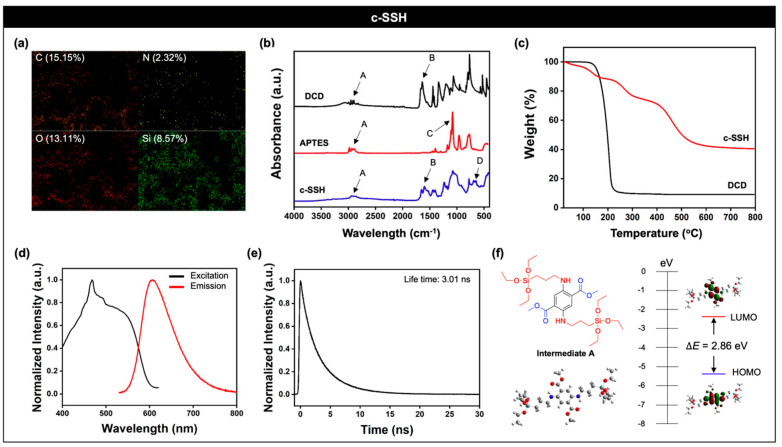
Material characterization and photophysical property analysis of **c-SSH**. (**a**) Energy-dispersive X-ray spectroscopy (EDS) element mapping images of **c-SSH**; carbon (C), nitrogen (N), oxygen (O), and silicon (Si). (**b**) Attenuated total reflectance-Fourier transform infrared (ATR-FTIR) spectra of DCD (black), APTES (red), and **c-SSH** (blue). A: ν(C–H), B: ν(C=O), C: ν(Si–O), D: δ(C–H, aromatic). Symbols: ν = stretching, δ = bending. (**c**) Thermogravimetric analysis (TGA) profiles of **c-SSH** (red) and DCD (black) under nitrogen atmosphere. (**d**) Excitation and emission spectra of **c-SSH** (solid-state) derived from 2D photoluminescence excitation (PLE) spectrum (Figure S1). Excitation wavelength: 400–700 nm. (**e**) Time-resolved fluorescence (TRF) signals of **c-SSH** in solid-state. Experiments were carried out using a time-correlated single-photon counting (TCSPC) method. The sample was excited using a 520 nm pulse and the sample TRF signals were collected at 610 nm. The average lifetime of **c-SSH** is 3.01 ns. (**f**) The optimized molecular structure, HOMO/LUMO, and the energy levels of **Intermediate A** by DFT calculations (B3LYP-d3/6-31+G(d)).

First, we characterized **c-SSH**. The element analysis, using energy-dispersive X-ray spectroscopy (EDS) showed that elements of **c-SSH** consisted of carbon (15.15%), nitrogen (2.32%), oxygen (13.11%), and silicon (8.57%) (Figure 2a). We then checked the finger-print peaks of DCD, APTES, and **c-SSH** in the attenuated total reflection-Fourier transform infrared (ATR-FTIR) spectrum (Figure 2b). Peaks at 3000–2800 cm^−1^ (A), which correlated with the stretching mode (ν) of C–H, were observed in DCD, APTES, and **c-SSH** [26]. DCD showed other finger-print peaks at 1700–1600 cm^−1^ (B), which also correlated with the ν(C=O) from the ester and ketone [27]. APTES showed a strong peak at 1105 cm^−1^ (C) as a ν(Si–O) [28,29]. The reaction of DCD and APTES, under the aerobic condition in the presence of Lewis acid (acetic acid), generated a benzene ring within the SBBF core, and we observed that **c-SSH** had finger-print peaks for the bending mode (δ) of aromatic C–H at 700–600 cm^−1^ (D) [30]. The SBBF generation and hydrolytic silica cross-linking formation were confirmed with thermogravimetric analysis (TGA) (Figure 2c). The DCD was vaporized below 200 °C, which corresponded with its boiling point (156 °C), but **c-SSH** showed a loss of weight, up to 800 °C, which represented the hybridization of the organic SBBF and inorganic silica.

With the material characterization results, we analyzed the photophysical properties of **c-SSH**. As expected, **c-SSH** showed a bright red emission under UV light (365 nm) in solid-state. The solid-state excitation and emission spectra were mainly obtained at 400–550 nm and 600–650 nm (Figure 2d), respectively. The excitation and emission spectra of **c-SSH** derived from the 2D photoluminescence excitation (PLE) spectrum and the 2D PLE spectrum were obtained by excitation at 400–700 nm (Figure S1). The excitation and emission spectra were obtained by integrating the 2D PLE spectrum at the excitation wavelength from 650–700 nm and 460 nm–510 nm, respectively. Time-resolved fluorescence (TRF) signals of **c-SSH** in solid-state were carried out using a time-correlated single-photon counting (TCSPC) method, and the average lifetime of **c-SSH** was 3.01 ns (Figure 2e, excitation: 520 nm, TRF signals collection: 610 nm). The quantum chemical calculation results of **intermediate A**, which was a silicon non-hybrid SBBF compound, showed 2.86 eV (=433 nm) of a HOMO-LUMO energy gap in the most optimized molecular conformation (B3LYP-d3/6-31+G(d) for the density functional theory (DFT) calculation), and it corresponded with the excitation spectra of **c-SSH** (Figure 2f). The electron density of **intermediate A** was more localized in the donor moiety (–NH–) in the HOMO; however, it was more localized in the acceptor moieties (–COOMe) in the LUMO. The SBBF was expected to undergo intramolecular charge transfer upon electronic excitation, such that the partial charge migrates from donor to the acceptor moiety.

### 3.3. Physicochemical Properties and Applications of **c-SSH**

**Figure 3:**
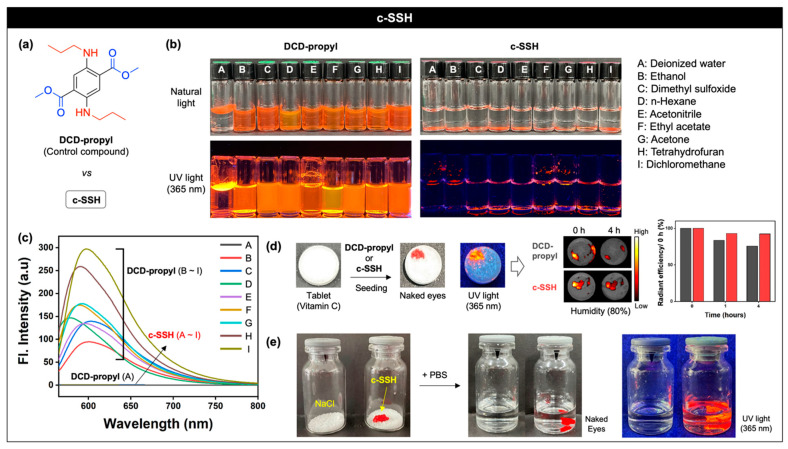
Physicochemical properties and applications of **c-SSH**. (**a**) Chemical structure of **DCD-propyl** (control compound). (**b**) Photos of **DCD-propyl** (32 mM) and **c-SSH** (10 mg/mL) in various solvents (A–I) under natural light and UV light (365 nm). **DCD-propyl** is dissolved in the solvents, and **c-SSH** is at the bottom of the vial. (**c**) UV/vis emission spectra of **DCD-propyl** (32 mM) and **c-SSH** (10 mg/mL) within various solvents (A–I) at 25 °C. Excitation wavelength: 477 nm. (**d**) Photos of vitamin C tablet with seeding of **DCD-propyl** (1 mg) and **c-SSH** (1 mg) under natural light and UV light (365 nm). Their fluorescence signal changed before and after incubation (4 h) under high-humidity conditions (80%). Excitation wavelength: 620–650 nm, emission channel: 690–740 nm. Graph (right): radiant efficiency plot from the fluorescence image (middle). Black bar: **DCD-propyl**. Red bar: **c-SSH**. (**e**) Photos of the vials containing NaCl (1 g) with/without **c-SSH** (**left**, 20 mg), and its solution after-treatment of the PBS (pH 7.4, 2 mL) under natural light (**middle**) and UV light (365 nm, **right**).

To identify the advantages of **c-SSH** in the materials view, we prepared an SBBF derivative (Figure 3a, named **DCD-propyl**, 1H NMR spectra: Figure S2) which had no triethoxysilane moiety, in comparison to the physicochemical property of **c-SSH**. **DCD-propyl** (10 mg/mL; 32 mM) and **c-SSH** (10 mg/mL) spiked with various solvents and their emission properties were analyzed (Figure 3b,c). **DCD-propyl** was soluble in most organic solvents, except deionized water (DI.H2O, floated), and showed a strong red emission. However, **c-SSH** did not dissolve in any solvents, and settled at the bottom of the vials. Such results represent that the silica coating shields the dissolution of the encapsulated SBBF moiety, and ensures the high stability and solvent tolerance of **c-SSH**.

Based on the high solvent stability of **c-SSH**, we attempted to explore the potential of **c-SSH** as an adjuvant to discern legitimacy from counterfeit medicines in tablets and injectables. First, we checked the humidity tolerance of **c-SSH** to use in tablets. For this analysis, a tablet of ascorbic acid (vitamin C) was prepared in which **DCD-propyl** (1 mg) and **c-SSH** (1 mg) were seeded respectively (Figure 3d). Under UV light **c-SSH** produced a red emission (365 nm) in the tablets, and we observed the emission changes for 4 h at 25 °C using a fluorescence imaging system (FTIS), under a high-humidity condition (humidity: 80%). The results showed that the emission signal of **c-SSH** within the tablet was maintained during the incubation, but **DCD-propyl** showed slightly decreased signals (76%), which indicated that **c-SSH** had a higher humidity tolerance than **DCD-propyl**. Next, we also confirmed that **c-SSH** could be used as an adjuvant to discern legitimacy from counterfeit medicines not only in tablet form but also in injection solutions. We put NaCl (1 g) into two vials as a model drug used in water, and added a piece of **c-SSH** into one of the two vials (Figure 3e). As expected, NaCl dissolved when phosphate-buffered saline (PBS, pH 7.4) was added, but **c-SSH** did not dissolve and emitted its original red fluorescence in the solution under UV light (365 nm). Considering that most organic dyes dissolve slightly in water, this property of **c-SSH** could be applied to ensure the authenticity of the drug. The emission property of **c-SSH** was also analyzed at a variety of temperatures and exposure conditions ranging from −196 °C to 250 °C (Figure S3). The emission signal of **c-SSH** was maintained at nearly all temperatures, except at 250 °C, where the SBBF moiety was carbonized. Overall, such results represent that **c-SSH** has a high tolerance under harsh conditions (high humidity, high temperature) and holds great potential for biomedical applications.

### 3.4. Material Characterization and Photophysical Properties of **n-SSH**

**Figure 4:**
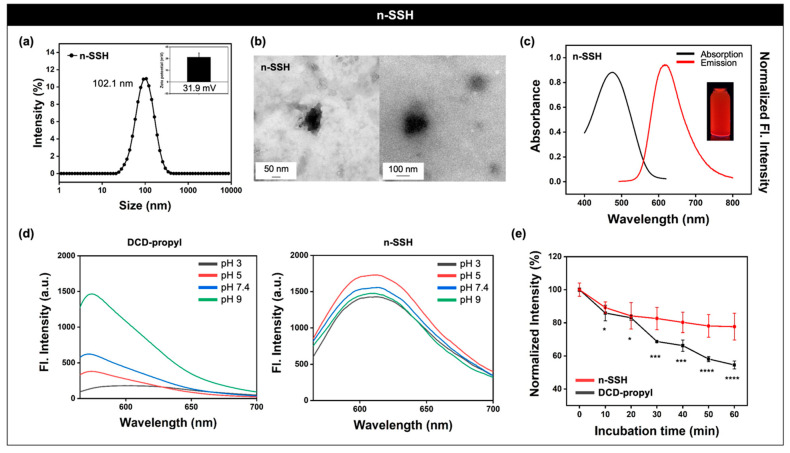
Material characterization and photophysical property analysis of **n-SSH**. (**a**) Average hydrodynamic diameter intensity distribution and zeta-potential value of **n-SSH** (1 mg/mL) in DI.H_2_O measured by dynamic light scattering (DLS). Polydispersity index (PDI): 0.314. (**b**) Transmission electron microscope (TEM) images of **n-SSH**. Scale bar: 50 nm (left), 100 nm (right). (**c**) UV/vis absorption and emission spectra of **n-SSH** (1 mg/mL) in DI.H_2_O. (**d**) Emission spectra of **DCD-propyl** (left, 3 mM) and **n-SSH** (right, 1 mg/mL) within various pH buffers at 25 °C. Excitation wavelength: 554 nm. (**e**) Emission intensity plot of **n-SSH** (1 mg/mL) and **DCD-propyl** (3 mM) as timed intervals (0–60 min) under UV light irradiation (3 W, 365 nm). Intensity was derived from the maximum wavelength (**n-SSH**: 613 nm; **DCD-propyl**: 579 nm). Excitation wavelength: 554 nm. The data are shown as the mean ± S.E.M. * *p* < 0.05, *** *p* < 0.001, **** *p* < 0.0001.

After the physicochemical property analysis of **c-SSH**, we then moved to the preparation and applications of nano-sized **SSH** (**n-SSH**). **n-SSH** was prepared in two steps: (i) physical grinding of **c-SSH** (Figure S4), and (ii) ultrasonic disintegration of grated **c-SSH** for 24 h within DI.H_2_O (20 mg/mL). The resulting **n-SSH** showed a similar element composition ratio of carbon, nitrogen, oxygen, and silicon compared with **c-SSH** (Figure S5). The particle size of **n-SSH** displayed a homogeneous average hydrodynamic diameter of 102.1 nm (polydispersity index; PDI: 0.3), and the zeta-potential was positive (31.9 ± 5.27 mV), due to the primary amine moiety from APTES (non-reacted residue) (Figure 4a). Transmission electron microscope (TEM) images of **n-SSH** showed a particle size of around 100 nm with a high uniformity (Figure 4b).

After the material characterizations of **n-SSH**, we identified the photophysical properties. **n-SSH** showed increased hydrophilicity to be dispersed in DI.H_2_O, and also showed the main absorption peak at 468 nm and an emission peak at 606 nm (Figure 4c), which were similar to the solid-state property of **c-SSH** (Figure 2d). Next, the pH-dependent emission property of **n-SSH** was analyzed and compared with **DCD-propyl**. **n-SSH** (1 mg/mL) and **DCD-propyl** (3 mM) spiked at various pH buffers (pH 3, 5, 7.4, 9) and there was no aggregation issue in the given concentrations. While the fluorescence intensity change of **DCD-propyl** was significant with the pH changes, **n-SSH** had emissions in the same wavelength (606 nm) with no considerable intensity decrement or peak shift, which represented the high pH tolerance of **n-SSH** (Figure 4d, Figures S6 and S7). To compare the light stability of **n-SSH** and **DCD-propyl**, they were placed under intense UV light (3 W) for 1 h, and their emission intensity was recorded every 10 min. Interestingly, the emission intensity of **DCD-propyl** gradually decreased to 50% at 1 h, but **n-SSH** had merely an approximately 20% decrease, which represented the higher photo-stability of the silica-hybridized **n-SSH** compared with the naked **DCD-propyl** (Figure 4e).

### 3.5. Biological Applications of **n-SSH**

**Figure 5:**
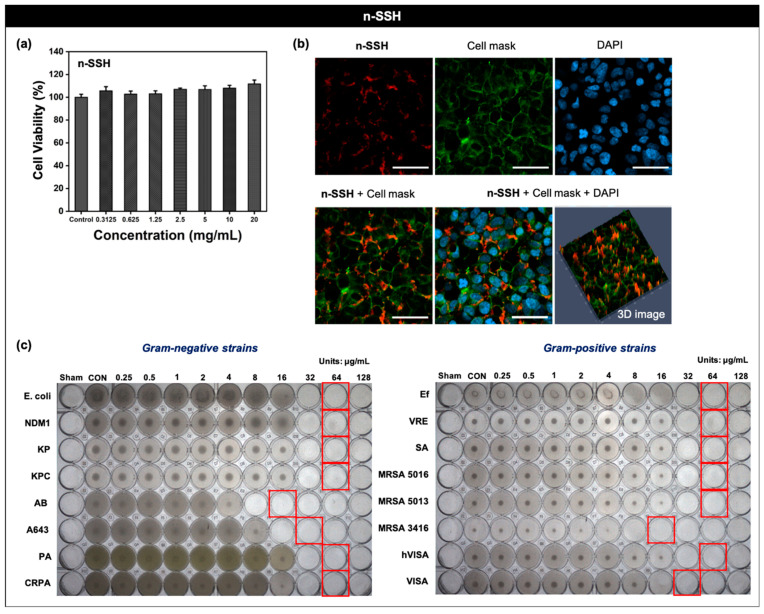
Biological applications of **n-SSH**. (**a**) Cell viability of the HEK293 cell line, after treating with **n-SSH** (0.3125–20 mg/mL), 24 h incubation period. The experimental results are shown as the mean ± S.E.M. (**b**) Confocal laser scanning microscopy (CLSM) images of HEK293 cells after treating with **n-SSH** (10 mg/mL), 6 h incubation period. Scale bar: 100 µm. Excitation and emission channel: red (561 nm, 576–700 nm), green (488 nm, 513–546 nm), and blue (405 nm, 410–450 nm). SA (serum albumin)-free media was used for incubation. (**c**) MIC assay results of **n-SSH** against 12 types of bacteria strains. **n-SSH** was serially diluted two-fold in a 96-well round-bottom microplate at different concentration ranges from 0.25 to 128 μg/mL. Incubation time: 24 h. Red box: minimum inhibitory concentration.

We further investigated the biomedical applications of **n-SSH**. The first application was a fluorescence-based bio-imaging of cells. Before confirming the bio-imaging ability of **n-SSH**, we evaluated the viability of the HEK293 (human embryonic kidney) cells after treatment with **n-SSH** (0–20 mg/mL) and incubation of 24 h, but there was no significant cellular toxicity at the given concentrations (Figure 5a). We conducted cellular imaging experiments with **n-SSH**, showing high biocompatibility and commercialized sub-organelle trackers (cell mask green plasma membrane tracker and DAPI). **n-SSH** (10 mg/mL) was treated to the HEK293 cells and incubated for 12 h at 37 °C, and the trackers were post-incubated for 20 min. Confocal laser scanning microscopy (CLSM) images showed that **n-SSH** appeared to be bound at the cellular membrane even after thorough PBS washing of the cells, and its signal increased in line with the incubation time (Figure 5b and Figure S8). The second application example of **n-SSH** was antibiotic effect analysis. We expected that the highly positive surface charge of **n-SSH** had antibiotic properties via bacteria membrane disruption [31], which is a well-known approach in the development of antibiotic materials. We treated **n-SSH** (0.25–128 μg/mL) to 16 types of Gram-positive and Gram-negative bacteria strains including multi-drug resistance (MDR) strains, and analyzed minimum inhibitory concentration (MIC) values. Ciprofloxacin (0.125–64 μg/mL) was used as a positive control [32], and dimethyl sulfoxide (DMSO) solvent and DCD (0.25 to 128 μg/mL; SBBF precursor) were used as the negative control. **n-SSH** and control compounds were serially diluted (two-fold) using CA-HMB broth in a 96-well microplate, and all bacterial suspensions were adjusted for turbidity to a 0.5 McFarland standard (1 × 10^8^ CFU/mL). The microplates were then incubated for 24 h at 37 °C. **n-SSH** showed an antibiotic effect in all types of bacteria at a concentration of 64 μg/mL or less (Figure 5c). The negative control showed no antibiotic effect in any strain (Figure S9).

## 4. Conclusions

In this report, we have disclosed a red-emitting SBBF-silica hybrid (**SSH**) material for the first time. It was revealed that the new formulation is able to be used in the as-prepared form (**c-SSH**) as well as the nano-sized form (**n-SSH**). We then systematically analyzed the physicochemical properties of **c-SSH** and **n-SSH**, comparing them with a non-hybrid SBBF compound, and confirmed that the **SSH** formulations have several advantages in terms of humidity tolerance, thermal stability, photo-stability, and environment insensitivity. We also applied **c-SSH** to use as an adjuvant to identify counterfeit medicines both in tablets and injection solutions, which holds a high potential for drug development. In addition, we successfully demonstrated the biological applications of **n-SSH**, including fluorescence imaging of cells and antibiotic assay. Based on our findings, we firmly believe that the **SSH** formulations could be applied in various fields as a unique dye-hybrid material.

## Data Availability

The data presented in this study are available in article or Supplementary Materials.

## Supplementary Materials

The following are available online at https://www.mdpi.com/article/10.3390/nano11082036/s1, Figure S1: 2D photoluminescence excitation (PLE) spectrum (λ_ex_: 400–700 nm) of **c-SSH**, Figure S2: ^1^H NMR of **DCD-propyl**, Figure S3: Thermal stability of **c-SSH**, Figure S4: Photos of the first step of the **n-SSH** preparation, Figure S5: Energy-dispersive X-ray spectroscopy (EDS) element mapping images of **n-SSH**, Figure S6: UV/vis absorption and emission of **n-SSH** and **DCD-propyl**, Figure S7: Absorption spectra of **DCD-propyl** and **n-SSH** in various pH buffers, Figure S8: CLSM images of HEK293 cells, Figure S9: MIC assay results of ciprofloxacin, DMSO, and DCD.
