# Supplementary material for: Red-Emitting SBBF (Single-Benzene-Based Fluorophore)-Silica Hybrid Material: One-Pot Synthesis, Characterization, and Biomedical Applications
Source: Nanomaterials (Basel). 2021 Aug 10;11(8):2036. doi: 10.3390/nano11082036 (PMC8399688; doi:10.3390/nano11082036)
Supplement: Supplementary file 1 [file nanomaterials-11-02036-s001.zip › nanomaterials-1326841-SI.pdf]

nanomaterials

Supplementary Materials

Red-emitting SBBF (Single-benzene-based fluorophore)-Silica Hybrid Material: One-pot Synthesis, Characterization, and Biomedical Applications

Jaehoon Kim [1], Jong Min An [1], Yuna Jung [1], Na Hee Kim [1], Youngwoong Kim [1], and Dokyoung Kim [1,2,3,4,5*]

[1] Department of Biomedical Science, Graduate School, Kyung Hee University, Seoul 02447, Republic of Korea; noblestylejay@gmail.com (J.K); jmahn1990@gmail.com (J.M.A); jungpeng159@gmail.com (Y.J); pionaheek@gmail.com (N.H.K); eeewq1111@gmail.com (Y.K); dkim@khu.ac.kr (D.K.)
[2] Department of Anatomy and Neurobiology, College of Medicine, Kyung Hee University, Seoul 02447, Republic of Korea; (D.K.)
[3] Center for Converging Humanities, Kyung Hee University, Seoul 02447, Republic of Korea; (D.K.)
[4] Medical Research Center for Bioreaction to Reactive Oxygen Species and Biomedical Science Institute, Kyung Hee University, Seoul 02447, Republic of Korea; (D.K.)
[5] KHU-KIST Department of Converging Science and Technology, Kyung Hee University, Seoul 02447, Republic of Korea; (D.K.)
* Correspondence: dkim@khu.ac.kr (D.K.)

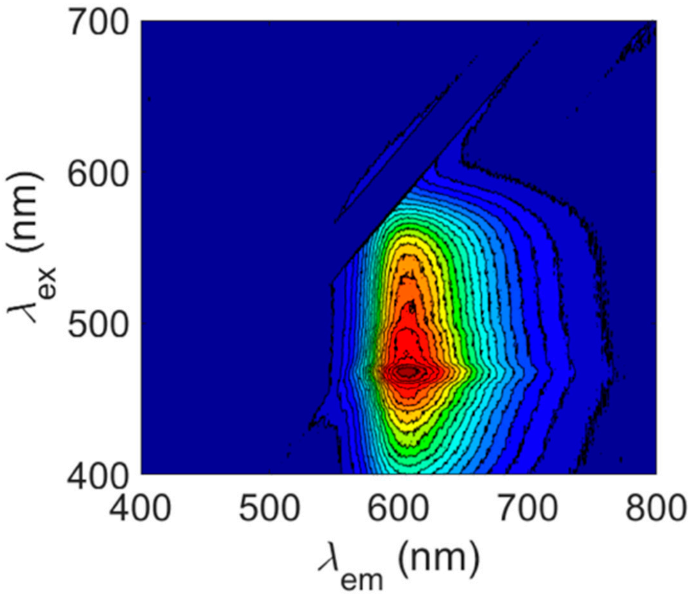

Figure S1. 2D photoluminescence excitation (PLE) spectrum (λ_{ex}: 400–700 nm) of **c-SSH**. X-axis: emission, Y-axis: excitation. Intensity: red (strong), blue (week).

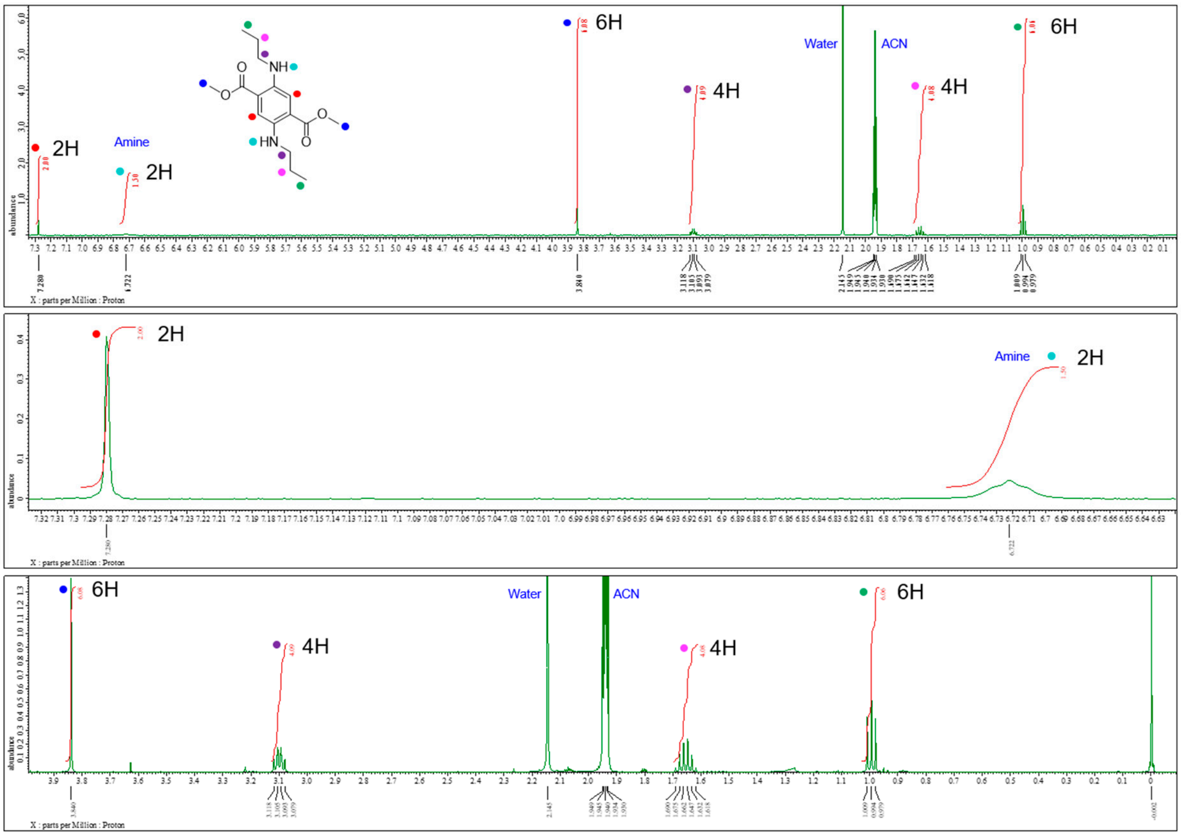

Figure S2. ^{1}H NMR of **DCD-propyl**. NMR solvent: Acetonitrile-d3.

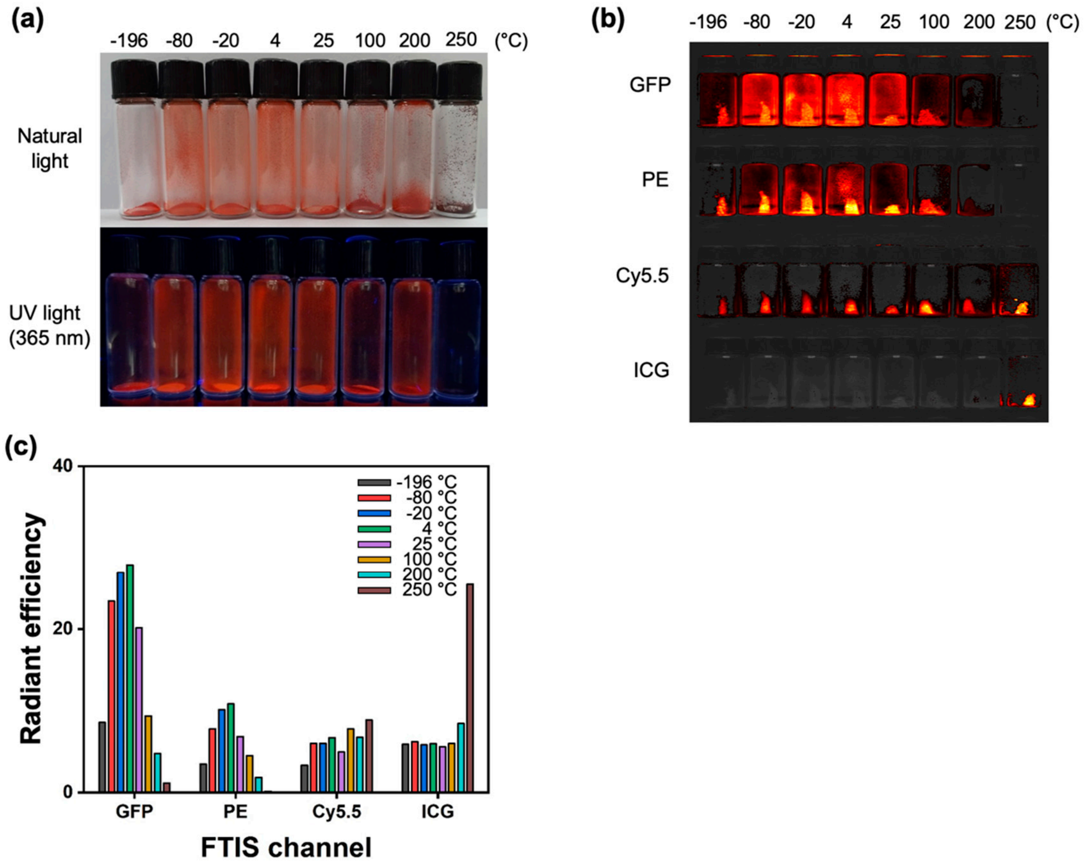

Figure S3. (**a**) Photos of the glass vial containing **c-SSH** at different temperatures under natural light (top) and UV light (365 nm, down). (**b**) FTIS images of the vials containing **c-SSH** at different temperatures and emission channels. Excitation and emission channel: GFP (390–490 nm, 500–550 nm), PE (530–570 nm, 575–640 nm), Cy5.5 (620–650 nm, 690–740 nm), and ICG (740–790 nm, 810–860 nm). (**c**) Radiant efficiency plot from panel (b). Heat exposure time: 10 min.

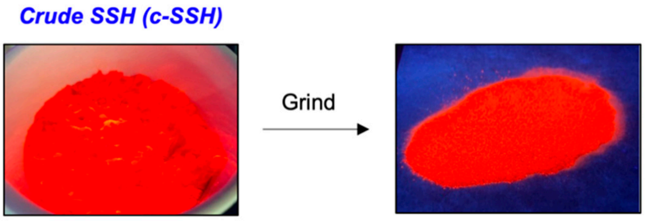

Figure S4. Photos of the first step of the **n-SSH** preparation. (left) Photos of **c-SSH** and (right) ground **c-SSH** under UV light (365 nm).

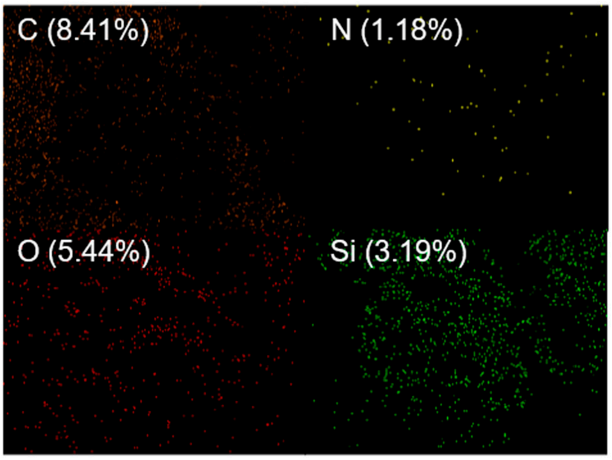

Figure S5. Energy-dispersive X-ray spectroscopy (EDS) element mapping images of **n-SSH** (C: 8.41%; N: 1.18%; O: 5.44%; Si: 3.19%).

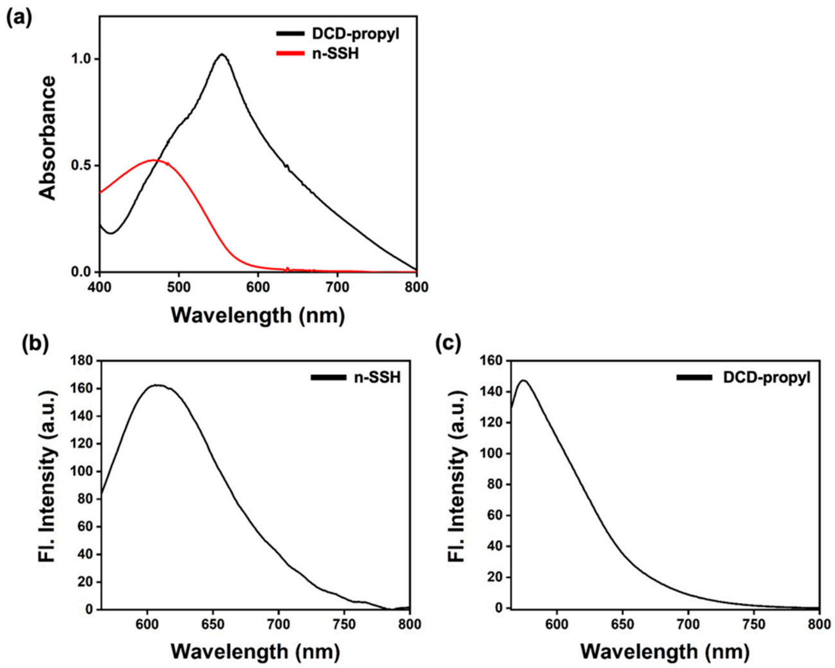

Figure S6. (**a**) UV/vis absorption and emission spectra of (**b**) **n-SSH** (1 mg/mL) in DI.H_2O (5% acetonitrile) and (**c**) emission spectra of **DCD-propyl** (3 mM) in DI.H_2O (5% acetonitrile). Excitation wavelength: 554 nm.

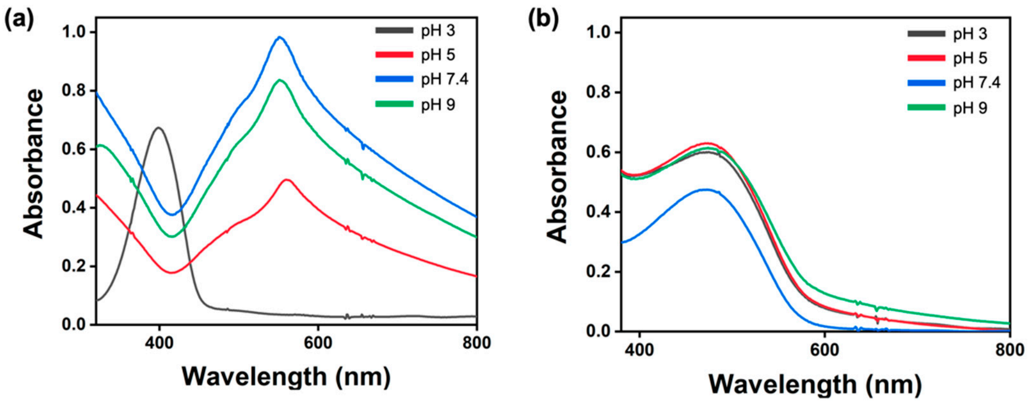

Figure S7. Absorption spectra of (**a**) **DCD-propyl** (3 mM) and (**b**) **n-SSH** (1 mg/mL) in various pH buffers (3, 5, 7.4, 9) at 25 °C.

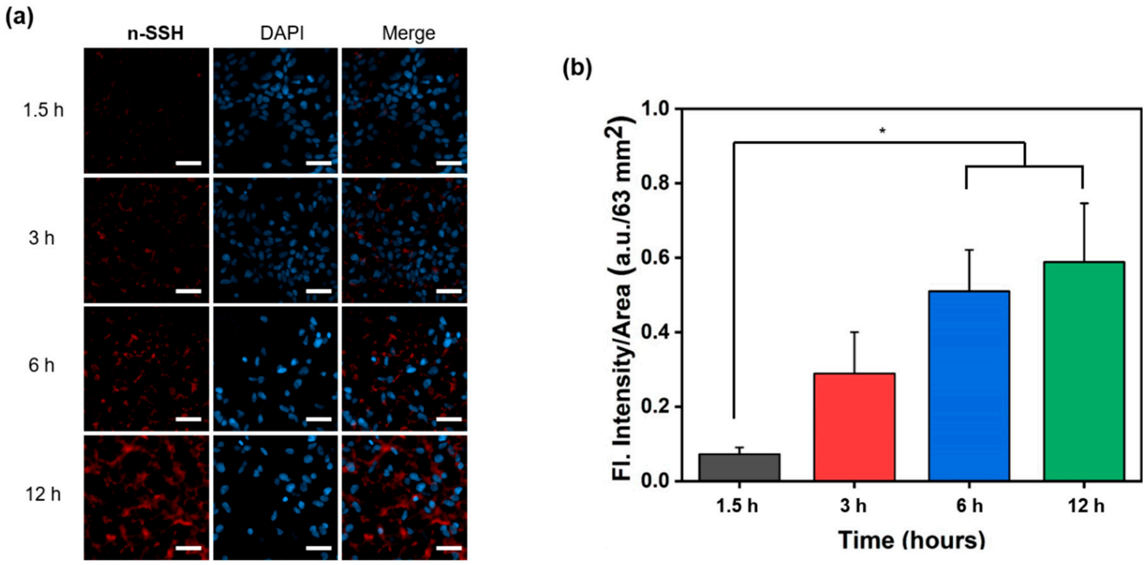

Figure S8. (**a**) CLSM images of HEK293 cells after being treated with **n-SSH** (10 mg/mL) and incubated for 1.5–12 h at 37 °C. Scale bar: 100 μm. Excitation and emission channel: red (561 nm, 576–700 nm), blue (405 nm, 410–450 nm). SA-free media was used for the incubation. (**b**) Fluorescence intensity plot from panel (a) (63 mm^2 areas in merge image) at given incubation time points. The data are shown as the mean ± S.E.M. *$p<0.05$.

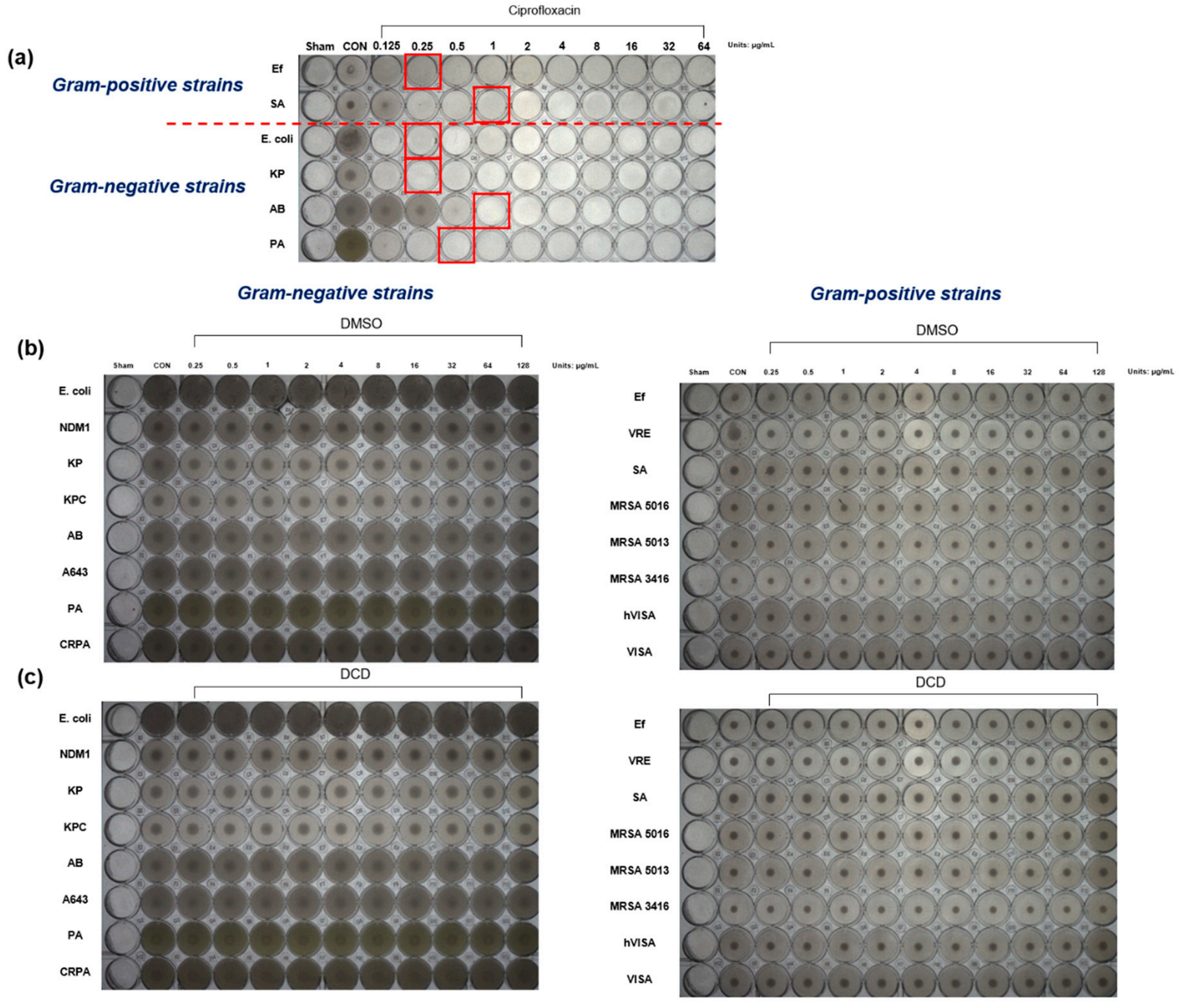

Figure S9. (**a**) MIC assay results of (**a**) ciprofloxacin (control) against 6 types of bacteria strains. Ciprofloxacin was serially diluted two-fold in a 96-well round-bottom microplate at different concentration ranges from 0.125 to 64 μg/mL. (**b**) MIC assay results of DMSO (control) and (**c**) DCD against 16 types of bacteria strains. DMSO and DCD were serially diluted two-fold in a 96-well round-bottom microplate at different concentration ranges from 0.25 to 128 μg/mL.
